# Ultrafast Laser Processing of Optical Fibers for Sensing Applications

**DOI:** 10.3390/s21041447

**Published:** 2021-02-19

**Authors:** Stephen J. Mihailov, Cyril Hnatovsky, Nurmemet Abdukerim, Robert B. Walker, Ping Lu, Yanping Xu, Xiaoyi Bao, Huimin Ding, Manny De Silva, David Coulas, Dan Grobnic

**Affiliations:** 1 National Research Council Canada, 100 Sussex Drive, Ottawa, ON K1A 0R6, Canada; Kyrylo.Hnatovsky@nrc-cnrc.gc.ca (C.H.); robert.walker2@nrc-cnrc.gc.ca (R.B.W.); Ping.Lu@nrc-cnrc.gc.ca (P.L.); Huimin.Ding@nrc-cnrc.gc.ca (H.D.); Manjula.DeSilva@nrc-cnrc.gc.ca (M.D.S.); David.Coulas@nrc-cnrc.gc.ca (D.C.); Dan.Grobnic@nrc-cnrc.gc.ca (D.G.); 2 Infinera Canada Inc., 555 Legget Dr., Ottawa, ON K2K 2X3, Canada; nabdukerim@infinera.com; 3 Center for Optics Research and Engineering, Shandong University, Qingdao 266237, China; yanpingxu@sdu.edu.cn; 4 Physics Department, University of Ottawa, 25 Templeton Street, Ottawa, ON K1N 6N5, Canada; Xiaoyi.Bao@uottawa.ca

**Keywords:** fiber Bragg gratings, random fiber gratings, femtosecond laser, fiber optic sensor

## Abstract

A review of recent progress in the use of infrared femtosecond lasers to fabricate optical fiber sensors that incorporate fiber Bragg gratings (FBG) and random fiber gratings (RFG) is presented. The important advancements in femtosecond laser writing based on the phase mask technique now allow through-the-coating (TTC) fabrication of Bragg gratings in ultra-thin fiber filaments, tilted fiber Bragg gratings, and 1000 °C-resistant fiber Bragg gratings with very strong cladding modes. As an example, through-the-coating femtosecond laser writing is used to manufacture distributed fiber Bragg grating sensor arrays for oil pipeline leak detection. The plane-by-plane femtosecond laser writing technique used for the inscription of random fiber gratings is also reviewed and novel applications of the resultant devices in distributed temperature sensing, fiber lasers and fiber laser sensors are discussed.

## 1. Introduction

High-power near infrared (IR) ultrafast laser systems, such as femtosecond (fs) regeneratively amplified Ti:sapphire lasers, proved themselves to be effective tools for machining of optical fibers for sensing applications [1]. The ultra-high intensity radiation that can be generated by these laser sources is effective in modifying dielectric materials either through ablative processes on the surface or by the induction of large index changes in the bulk. Focused beam intensities of over 10^13^ W/cm^2^ are easily realized and are sufficient to initiate nonlinear light absorption in the optical fiber glass during the laser pulse [2,3]. This highly localized energy deposition produces an electron plasma within the focal volume which after several picoseconds transfers its energy to the bulk material. This, in turn, leads to permanent material modification such as compaction and/or defect formation, localized melting, and formation of nanogratings [4,5] or voids [6,7]. The specific kind of material modification depends on the intensity of the laser pulse and other laser irradiation conditions.

Over the past two decades, fs laser systems have been extensively used to manufacture temperature and strain sensors based on fiber Bragg gratings (FBG) [8], improved optical frequency domain reflectometers (OFDR) using fibers with randomly positioned scattering sites [9], complex bend sensors based on waveguide inscription in fiber claddings [10,11], or for direct machining of innovative biosensors [12].

This review paper will be divided into two broad themes. First, we will summarize recent work from our laboratories on fs laser processing of optical fibers to produce FBGs directly through protective polymer coatings of fibers using the phase mask technique and how the resultant devices have been applied for environmental sensing. Second, we will discuss our recent advances in engineering random fiber gratings (RFGs) using fs lasers and the plane-by-plane technique and how these fiber optic devices have been implemented into the design of light sources and sensors.

## 2. Through-the-Coating Fiber Bragg Gratings

### 2.1. Ultraviolet vs. Infrared Femtosecond Irradiation

Optical filter technology based on FBGs is well established in the fields of telecommunications and sensing. Originally, FBGs were manufactured by exposing the germanium-doped (Ge-doped) core of silica (SiO_2_) telecommunications fiber to periodically modulated high intensity ultraviolet (UV) laser radiation. In such cases, the induced index change and modulation pattern in the core of the fiber results from color center formation [13] and localized compaction of the glass matrix [14]. From a manufacturing perspective, grating inscription with UV lasers is limited to Ge-doped fibers that often need to be photosensitized by processes such as hydrogen loading (H_2_-loading), or the presence of extremely high Ge-concentrations in the silica fiber core. Furthermore, because protective polymer coatings on commercially available optical fibers typically absorb UV light, coating removal and reapplication before and after grating inscription, respectively, become necessary processing steps. Finally, stability of the photo-induced index change has to be assured by annealing the device at temperatures above its normal operating temperature. In this way, unstable color centers that contribute to the overall index change are removed [15].

For fs-laser-written FBGs (fs-FBG) the nature of induced index change is different [4,5,6,7]. Gratings can be fabricated with fs lasers in any optical fiber that is transparent to the low-signal IR light and are therefore not reliant on the presence of a specific dopant. The inscription process does not require enhancement of fiber photosensitivity, such as by H_2_-loading, and can be carried out directly through protective polymer coatings. All of the exotic Bragg grating structures that have been fabricated with UV laser systems in photosensitive telecommunications fibers, such as chirped gratings, phase shifted gratings, apodized gratings, gratings with suppressed cladding modes, and tilted or blazed gratings, can also be fabricated with fs lasers using similar processes.

### 2.2. Point-by-Point Femtosecond FBG Inscription Process

Fs-FBGs have been fabricated by either using the point-by-point (PbP) technique or the phase mask technique. The PbP approach involves the focusing of single fs laser pulses into the core region of the fiber using a high numerical aperture (high-NA) microscope objective. Each pulse makes highly localized changes to the refractive index that are seen by the propagating mode(s) in the optical fiber as a FBG ‘plane’. Points are created sequentially in a step-and-repeat fashion by translating the beam using high resolution mechanical translation stages [16]. Through-the-coating (TTC) FBG inscription was first demonstrated using the PbP technique [17]. The advantages of this approach come from the versatility of the grating structure that can be created as it relies solely on the positioning of the focal spot within the fiber and control of the precision translation. Because the fs pulse is highly focused, lower power fs sources can be used, such as high-power oscillators or femtosecond fiber laser sources rather than a regeneratively amplified source. The major disadvantage of this approach is that it is not well-suited for mass production of identical structures.

### 2.3. Phase Mask Technique for Femtosecond FBG Inscription

The phase mask is a diffractive optical element, specifically a transmission diffraction grating. It is especially made using microfabrication techniques whereby the grating pattern, produced either holographically or by electron beam lithography, is precisely etched into a silica substrate. To minimize the transmission of zero order diffraction, the etch depth of the grating pattern is approximately equal to the laser wavelength that will be used to illuminate the mask and create the Bragg grating pattern in a fiber [18]. Despite its seeming simplicity, the phase mask technique exhibits several characteristic effects that originate from the presence of a strongly dispersive optical element (i.e., the phase mask) in the beam path. Some of these effects will be briefly discussed in the following subsections.

The use of a phase mask for FBG inscription using UV lasers, such as KrF excimer laser systems, has been very successful from an industrial perspective [13]. The phase mask automatically matches path lengths of diffracted interfering beams (±1 diffraction orders) significantly reducing alignment tolerances as compared to bulk interferometers. This aspect is especially important when FBG inscription is performed with (i) excimer laser systems that have spatial coherence on the order of ~100 μm, which requires the path lengths of interfering beams to be matched to that level or (ii) fs lasers, which also requires similar levels of path length matching, e.g., 30 μm for a 100 fs pulse. Even though the phase mask technique generally allows for mass production of identical FBGs, there are several additional factors that need to be considered when fs lasers are used instead of nanosecond or continuous wave UV lasers for FBG inscription.

**Figure 1:**
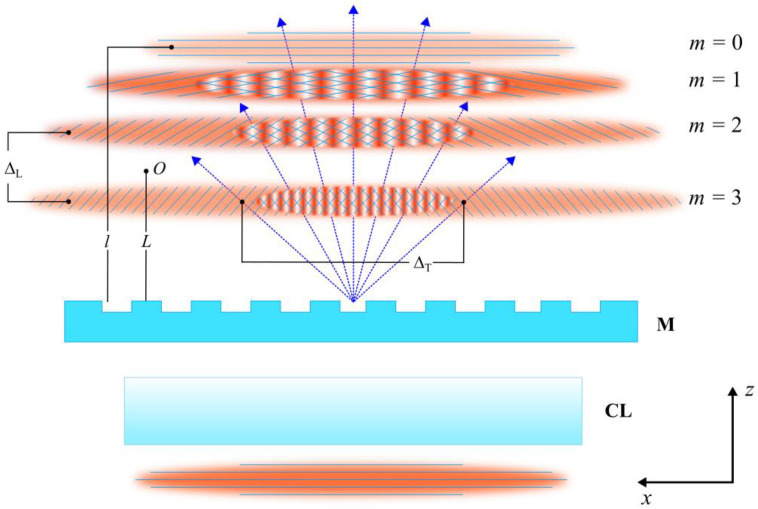
Interference of fs pulses diffracted by a phase mask that produces four diffraction orders (*m* = 0 to 3). **M** denotes the phase mask, **CL** is the cylindrical lens, Δ_T_ is the transverse walk-off, Δ_L_ is the longitudinal walk-off, *L* is the distance from **M** to the observation point (*O*), *l* is the distance from **M** to the pulse front of the 0th diffraction order. The pulse phase fronts are normal to the propagation direction of the respective diffraction orders [20].

For the FBG inscription process used in our experiments, the laser source was a *Coherent Libra* Ti:sapphire regenerative amplifier operating at a wavelength *λ*_0_ = 800 nm and a repetition rate of 1 kHz. The Fourier transform limited pulse duration of this laser source was 80 fs which corresponds to a spectral bandwidth of Δ*λ* = 12 nm. The output beam radius (at the 1/e^2^-intensity level) of the laser source was *w*_0_ ~3.5 mm. The exposure setup shown schematically in Figure 1, used a holographically patterned reactive ion etched silica phase mask that was precision etched to minimize zero order transmission at 800 nm. According to the manufacturer (*Ibsen*), the mask had a period *d* = 1.071 μm with a 0.01 nm period accuracy and uniformity. The thickness of the *Ibsen* mask substrate was *t =* 2.1 mm. The laser beam was focused with plano-convex acylindrical lenses (from *Asphericon*) made of OHARA S-LAH64 glass. The focal length of the lenses was either *f* = 12 mm or *f* = 15 mm with the respective clear aperture being 15 mm × 15 mm and 18 mm × 18 mm. The curved surface was designed to correct spherical aberration in one dimension. Transmission spectral measurements of fabricated FBGs were taken using a tunable laser and power meter with a spectral resolution of 10 pm.

#### 2.3.1. Longitudinal and Transverse Walk-Off

When a fs pulse is focused by a cylindrical lens and then passes through a phase mask at normal incidence, it is diffracted at angles *θ_m_* = sin^−1^(*mλ*_0_/*d*), where *λ*_0_ is the central wavelength of the fs pulse, *d* is the mask pitch and *m* is the diffraction order number. The phase fronts of the resulting diffracted fs pulses are normal to their propagation direction. The short time duration of the laser pulse results in longitudinal walk-off Δ_L_ [19] of the diffracted pulses belonging to different diffraction orders as they propagate away from the mask, see Figure 1 [20].

At a distance *L* from the mask, a pure two-beam interference pattern produced by the +*m* and −*m* diffracted orders will be observed when the following condition is satisfied [20]:

(1) $$L\geq \frac{\lambda_{o}^{2}}{\Delta \lambda }\frac{\cos\left(\sin^{-1}\left(m\lambda_{o}/d\right)\right)}{\cos\left(\sin^{-1}\left(\left(m-1\right)\lambda_{o}/d\right)\right)-\cos\left(\sin^{-1}\left(m\lambda_{o}/d\right)\right)}$$

where Δ*λ* is the FWHM bandwidth of the fs pulse and *m* is assumed to be positive. Equation (1) can be derived by equating the longitudinal walk-off L of the femtosecond pulses diffracted into different orders and the FWHM of the field autocorrelation function of the pulses. The resulting interference pattern for the ±1 orders has a pitch that is half of that of the phase mask, i.e., *d*/2. Near the mask, interference of multiple orders (0, ±1, ±2, etc.) creates a Talbot pattern [19,21,22,23]. There is also transverse walk-off Δ_T_ of the laterally overlapping ±*m* orders, which at a distance *L* from the mask is given by Δ_T_ = 2 *L* tan (*θ_m_*).

#### 2.3.2. Focal Spot Minimization

In addition to the above geometrical effects of the interaction between the fs pulse and the phase mask, chromatic diffraction and aberration effects need to be considered, especially in the context of tight focusing geometries and small-pitch phase masks. The impacts of these effects are rigorously analyzed by Abdukerim et al. [20]. It should be noted that for the experiments in [20] the authors introduced an interference filter in the beam to reduce the original Δ*λ* from 12 nm to 6 nm.

The impact of angular chromatic dispersion of the broadband fs pulse by the phase mask and chromatic aberration of the cylindrical lens used to focus the pulse onto the fiber core results in an elongation of the focal spot along the optical axis. It was shown [20] that this elongation (Δ*z_cm_*) can be expressed as:

(2) $$\Delta z_{cm}=\frac{m^{2}\lambda_{0}L\Delta \lambda }{d^{2}-m^{2}\lambda_{0}^{2}}$$

**Figure 2:**
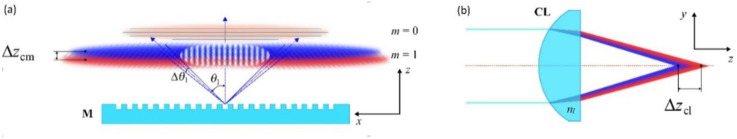
Focal elongation caused by chromatic dispersion of the mask Δ*z_cm_* (**a**) and chromatic aberration of the cylindrical lens Δ*z_cl_* (**b**). In (**a**), Δ*θ*_1_ is the angular spread of the spectrum of the 1st diffraction order corresponding to a pulse bandwidth Δ*λ*. For clarity, only the 0th and 1st diffraction orders are considered. Please note that ‘red’ light is focused closer to **M** in (**a**) and farther from **M** in (**b**).

This results in the ‘red’ light focus appearing closer to the phase mask than the ‘blue’ light focus. The cylindrical lens also introduces focal elongation but, in this case, the ‘blue’ spectral component of the pulse is focused closer to the mask than the ‘red’ component. The elongation of the focus after the phase mask due to chromatic aberration of the lens (Δ*z_cl_*) can be expressed as [20,24]:

(3) $$\Delta z_{cl}=-\frac{f\cos\left(\theta_{m}\right)}{n_{l}-1}\left(\frac{dn_{l}}{d\lambda }\right)\Delta \lambda$$

where *f* is the focal length of the lens and *n_l_* is the refractive index of the lens. Thus, Δ*z_cm_* can in principle be counteracted by Δ*z_cl_* (see Figure 2 [20]).

In cases where tight focusing of the fs laser beam is required, such as for TTC inscription of FBGs [25,26,27,28], additional effects resulting from conical diffraction of the mask and spherical aberration introduced by the mask substrate need to be considered.

**Figure 3:**
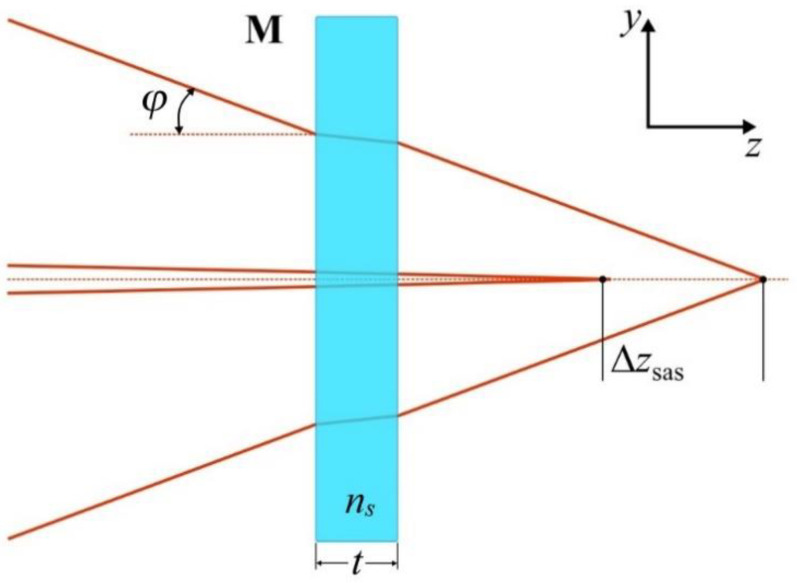
Focal elongation Δ*z_sas_* caused by the plane parallel mask substrate. Please note that marginal rays are focused farther from the mask than paraxial rays.

Abdukerim et al. showed that the focal elongation due to spherical aberration introduced by the phase mask substrate can be written after the phase mask as [20]:

(4) $$\Delta z_{sas}=\frac{\left(n_{s}^{2}-1\right)t\cos\left(\theta_{m}\right)\varphi^{2}}{2n_{s}^{3}}$$

where *n_s_* is the refractive index of the phase mask substrate, *t* is the mask thickness and *φ* is the angle of focused marginal rays’ incident on the phase mask (see Figure 3).

When tight cylindrical focusing of the beam incident on the mask is considered, paraxial rays lie in a plane that is perpendicular to the mask grooves (i.e., in-plane diffraction). Marginal rays, however, are no longer perpendicular to the grooves of the mask resulting in off-plane or conical diffraction. The result is that for a given diffracted order *m*, the marginal rays are focused closer to the mask than the paraxial rays. It can be shown that the focal elongation due to conical diffraction can be expressed as [20]:

(5) $$\Delta z_{cdm}=\frac{Lm^{2}\lambda_{0}^{2}\varphi^{2}}{2\left(d^{2}-m^{2}\lambda_{0}^{2}\right)}$$

**Figure 4:**
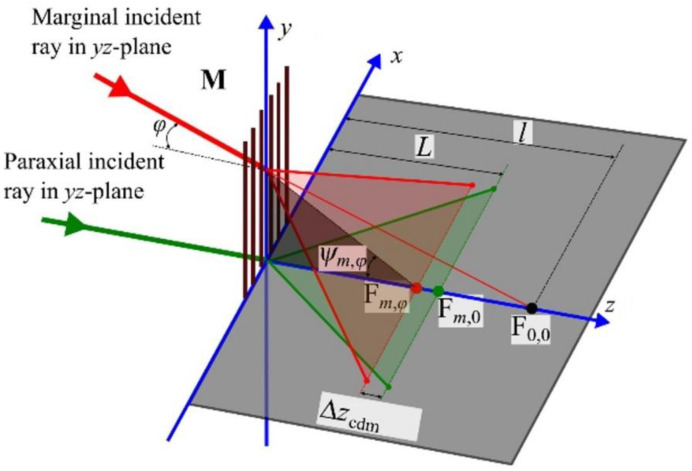
Ray propagation and the focal elongation Δ*z_cdm_* caused by conical (off-plane) diffraction. Please note that the marginal focus (i.e., F*_m_*_,*φ*_) lies closer to the mask than the paraxial focus (i.e., F*_m_*_,0_). Compare with Figure 3.

The focal elongation Δ*z_cdm_* is depicted schematically in Figure 4. As in Figure 3, the angle of the marginal rays is denoted by *φ*. In Figure 4, F_0,0_ denotes the paraxial focus of the 0th diffraction order, F*_m_*_,0_ and F*_m_*_,*φ*_ respectively denote the paraxial and marginal foci of the *m*th diffraction order, *L* and *l* respectively denote the distance from **M** and F*_m_*_,0_ (observation point *O* coincides with F*_m_*_,0_) and the distance from **M** to F_0,0_. For simplicity, only the 0th and 1st diffraction orders are considered. Please note that the marginal focus (i.e., F*_m_*_,*φ*_) lies closer to the mask than the paraxial focus (i.e., F*_m_*_,0_). This is opposite to the case of spherical aberration caused by the phase mask substrate.

The distance *L*_1_ at which chromatic dispersion of the mask is exactly balanced by chromatic aberration of the cylindrical lens is given by:

(6) $$L_{1}=\frac{-f}{n_{l}-1}\frac{dn_{l}}{d\lambda }\frac{{\left(d^{2}-m^{2}\lambda_{0}^{2}\right)}^{3/2}}{m^{2}\lambda_{0}d}$$

which is derived by equating Δ*z_cm_* (Equation (2)) and Δ*z_cl_* (Equation (3)). For a given *m* and *λ*_0_, *L*_1_ is solely determined by the mask and lens parameters and does not depend on, i.e., it will be the same for any laser source. Similarly, the distance *L*_2_ at which spherical aberration introduced by the mask substrate is exactly balanced by conical (off-plane) diffraction of the mask can be derived by equating Δ*z_sas_* (Equation (4)) and Δ*z_cdm_* (Equation (5)). In the 3rd-order approximation with respect to the focusing angle *φ* (*φ* < 0.3 rad) the condition Δ*z_sas_* = Δ*z_cdm_* yields:

(7) $$L_{2}=\frac{n_{s}^{2}-1}{n_{s}^{3}}\frac{{\left(d^{2}-m^{2}\lambda_{0}^{2}\right)}^{3/2}t}{m^{2}\lambda_{0}^{2}d}$$

As it can be seen from Equation (7), *L*_2_ does not depend on *φ*, i.e., it will be the same for any focusing cylindrical lens provided that its numerical aperture NA is less than 0.3.

Thus, there are two independent sets of phenomena that affect the spreading of the focal spot: (i) angular chromatic dispersion originating from the mask (Equation (2)) which is counteracted by chromatic aberration of the cylindrical focusing lens (Equation (3)) and (ii) spherical aberration caused by the mask substrate (Equation (4)) which is counteracted by conical diffraction (Equation (5)).

The interaction of these effects is especially important to consider when TTC inscription of FBGs is being performed [28]. In such an instance, tight focusing of the fs IR beam is required in order to ensure a large differential in light intensity seen by the fiber core and the fiber coating [25]. Furthermore, the highest reflectivity gratings are created when the resulting grating in the fiber has a period that is at the fundamental Bragg resonance [19], i.e., *d*/2. Phase masks used to create such a resonance at telecommunications wavelengths in silica fibers using IR beams have large 1st-order diffraction angles (more than 48° for a 1.071 μm-pitch mask). In this case, the position from the mask where a distinctive maximum in the focal peak intensity is observed will critically depend on the mask substrate (thickness, refractive index), focusing cylindrical lens (focal distance, refractive index), and diffraction angle of the mask (mask period).

**Figure 5:**
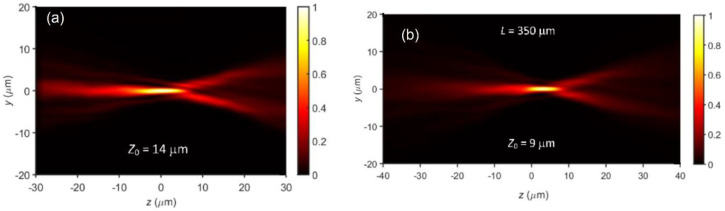
Focal intensity distributions in the yz-plane of (**a**) the beam from the regenerative amplifier using the 12-mm-focal-length acylindrical lens whose effective numerical aperture is sin(*φ*) = 0.26 and of (**b**) when the beam is focused through the 1.071 μm pitch, 2.1 mm thick phase mask. Distance between the focal spot and the phase mask surface is 350 μm. In (**a**,**b**), the beam is propagating from left to right. [20].

To estimate the quality of the focusing optics and output beam from the regenerative amplifier, 3D-time averaged intensity distributions measurements about the focal region were performed using the technique described in [23]. Briefly, the respective xy-intensity distributions with a 1 μm separation along the *z*-axis were projected onto a Complementary Metal Oxide Semiconductor (CMOS)matrix by means of a high numerical aperture (i.e., NA = 0.9) objective lens, recorded and combined into 3D stacks. The yz-intensity distributions shown in Figure 5 were obtained by averaging the values of points with fixed (*y_i_*, *z_i_*)-coordinates along the *x*-axis and projecting the respective mean values onto the yz-plane. Figure 5a) shows the focal intensity distribution of the output beam in the yz-plane when the beam is focused with the 12 mm-focal-length acylindrical lens described in Section 2.3. Considering free space Gaussian beam optics only, the focal spot line width 2*w* ≈ 2*λ*_0_*f/πw_o_*, For a collimated beam with diameter, *w_o_* = 3.5 mm, *λ*_0_ = 800 nm and *f* = 12 mm, the resulting focal spot would be 1.75 μm. From Figure 5a) the line width of the focal spot is 1.9 ± 0.1 μm, which is consistent with the Gaussian beam calculation.

**Figure 6:**
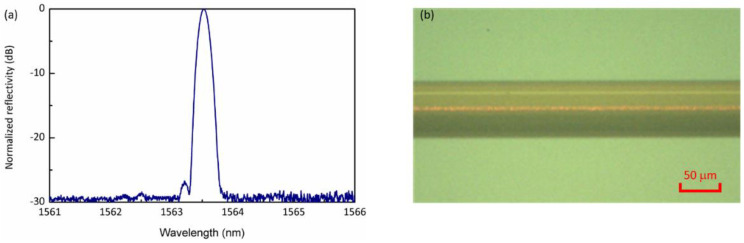
(**a**) Reflection spectrum (~6 dB in transmission) of an FBG written in a 50 μm diameter fiber through the protective polyimide coating. (**b**) An optical microscopy image of the 50 μm fiber containing the FBG. The refractive index modulation of the FBG is ~1.5 × 10^−4^. To visualize the FBG, red light at 637 nm was coupled into the fiber core [23].

Considering the laser writing conditions used in Ref. [20] (i.e., *λ*_0_ = 800 nm, Δ*λ* = 6 nm, *f* = 12 mm, *n*_l_ = 1.776, d*n_l_*/d*λ* = −0.0371 μm^−1^; *d* = 1.071 μm, *t* = 2.1 mm, *n*_s_ = 1.453). The bandwidth of the 80 fs pulses (i.e., 12 nm) was narrowed down to 6 nm by means of a narrowband filter. The minimal focal spot elongation and maximum peak intensity occurred at *L* ~350 μm from the mask and is shown in Figure 5b. The effect at this *L* was very pronounced and, for the first time, low-loss FBGs could be directly inscribed inside a non-photosensitized polyimide-coated fiber 50 μm in diameter (see Figure 6). Previously, the phase mask approach to fs-FBG inscription through polyimide coatings of Ge-doped 50 μm fibers was only possible when the fiber was photosensitized using high-pressure deuterium loading [29].

All in all, the experimental results agree well with the predictions and estimates based on the above analysis, where the chromatic effects are presented in terms of just two different wavelengths (i.e., ‘blue’ and ‘red’) and monochromatic aberrations are introduced as ray optics phenomena. The limitations of the formalism are obvious (i) as it provides only qualitative information about the intensity distribution in the line-shaped focal volume and (ii) neglects temporal pulse distortions caused by the rather complex optical setup consisting of an acylindrical lens, a plane-parallel plate, and a phase mask. To calculate the temporal and spatial distribution of the electric field in the focal volume, diffraction needs to be taken into account. We also note that a fully rigorous treatment of the problem should include the electromagnetic diffraction of light focused through the highly curved cylindrical surface of the fiber, consider residual aberrations of the acylindrical lens, and take into account beam imperfections, which are usually characterized by the beam quality factor (i.e., M^2^-factor). In this respect, our simple semi-quantitative formalism reinforced by intensity distribution measurements after the mask (Figure 5b) provides a straightforward and highly intuitive approach to identify optimum laser writing conditions to fabricate FBGs using the phase mask technique.

#### 2.3.3. Tilted TTC-FBGs

Tilted FBGs are Bragg grating structures where the grating planes are not normal but tilted with respect to the fiber axis. Light propagating in the guided mode can then couple into backward propagating cladding modes or even radiation modes depending on the tilt angle. Enhanced coupling to cladding modes with tilted Bragg gratings have been used to fabricate multi-parameter sensors, because the cladding modes respond differently to fiber strain as compared to the core mode making a combined strain-temperature sensor possible [30]. Until recently [31], tilted FBGs could be inscribed through protective polymer coatings only using a variation of the PbP technique with fs lasers [32]. In this respect, the TTC inscription of tilted FBGs using the phase mask technique would be advantageous from a sensor manufacturing perspective. In the absence of a fiber coating, fs lasers and phase masks were used to write tilted FBGs by translating the fiber vertically and along the fiber axis simultaneously while the fiber was in the beam focus [33]. In this instance, larger pitched phase masks that produced higher order Bragg gratings were used.

**Figure 7:**
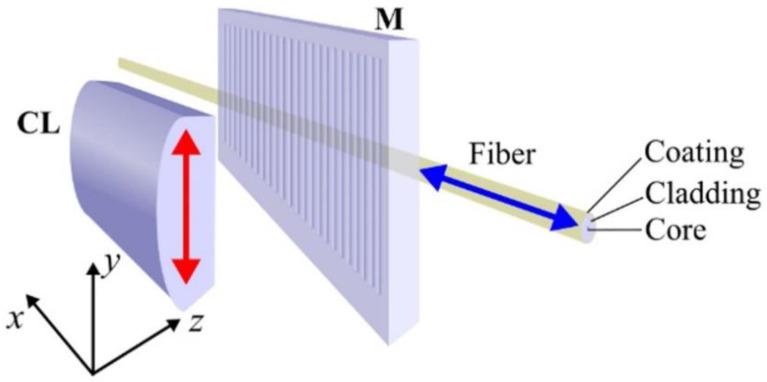
Experimental setup for writing tilted FBGs.

The TTC tilted FBGs were written using the experimental conditions given in Section 2.3. The fs laser beam was expanded along the *x*-axis and focused through the *Ibsen* mask (**M** in Figure 7) using an acylindrical lens with a focal length *f* = 15 mm (**CL** in Figure 7). The linear polarization of the fs laser beam was oriented parallel to the mask grooves. Polyimide-coated Corning SMF-28 was placed along the line-shaped focus and aligned parallel to **CL**. The fiber was positioned at *L* ~300 μm away from the phase mask, which was the optimal distance taking into account diffraction and dispersion effects discussed previously. Laser intensities used resulted in the formation of Type I index change [34]. The position of the laser line focus on the fiber core was aligned by using the techniques of nonlinear photoluminescence microscopy and dark-field microscopy [23]. The piezo-actuated stages for translating **CL** and the fiber were triggered by the same signal generator through a variable power splitter to control the translation ratio along the *x*- and *y*-axis. The inscription set-up for TTC tilted gratings is shown in Figure 7.

Since the fiber itself acts as a cylindrical lens, the refraction of the converging fs laser beam at the fiber surface has to be taken into account when calculating the actual translation range of the focus inside the fiber. For all the tilted FBGs, the vertical displacement Δ*L* of the fs laser beam along the *y*-axis was kept at 15 μm to cover the entire core. Under the small-angle approximation, the transverse movement Δ*Y* of the focus inside the fiber is then given by Δ*Y* ≈ Δ*L*/*n* (*n* is the refractive index of the fiber) [31], which results in Δ*Y* ~10 μm. The period of grating planes along the fiber axis is still given by *d*/2. Hence, tilted FBGs fabricated with different tilt angles are expected to have their main Bragg resonances positioned at the same wavelength. The transmission spectra of tilted FBGs were monitored with a spectral resolution of 0.01 nm throughout the writing process using a tunable laser and a power meter.

**Figure 8:**
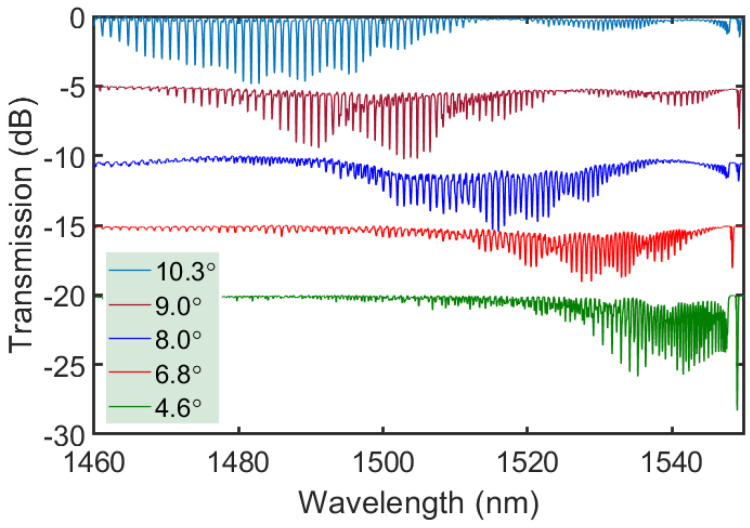
Transmission spectra of tilted FBGs written through polyimide coating of SMF-28 with different tilt angles of 4.6°, 6.8°, 8.0°, 9.0°, and 10.3°.

The transmission spectra of TTC-written tilted FBG with different tilt angles up to 10.3° are shown in Figure 8. In each case, the cladding modes reach ~5 dB in transmission and gradually shift to shorter wavelengths as the tilt angle increases. The cladding modes appear to have an irregular shape due to the presence of the polyimide coating, which has a higher refractive index than silica. The slight variation of the Bragg wavelength is caused by the difference in tension applied while loading the fiber into the fiber-holding jig and then releasing the fiber after the inscription. It is noted that when tilt angles are under 4°, the cladding modes are confined to within a ~20 nm wavelength range near the Bragg resonance, allowing for the potential to concatenate multiple tilted FBGs in a distributed sensing architecture.

**Figure 9:**
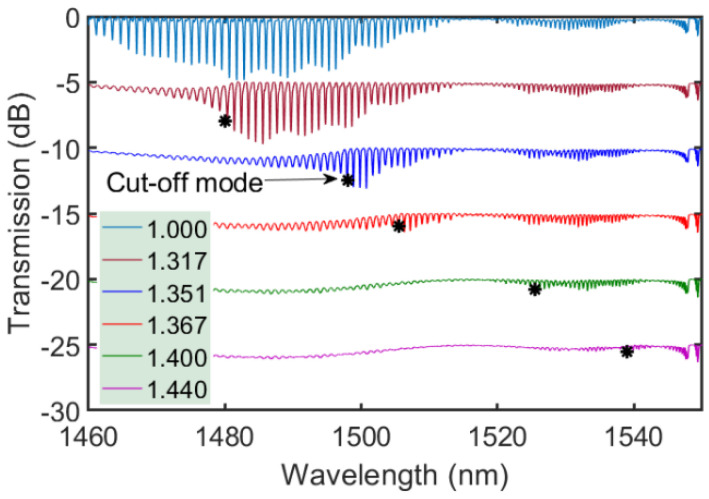
Transformation of the transmission spectra of a 10.3° tilted FBG written through the coating in response to different surrounding refractive indices.

With only a thin polyimide coating of 10 μm, the tilted FBGs can still be used to detect changes in the refractive index of the environment surrounding the grating. Figure 9 shows the variation in transmission spectra of a polyimide coated 10.3° tilted FBG as a function of local refractive index at room temperature.

The accuracy of determining the cut-off wavelength is ±0.7 nm within the spectral range of 1460–1510 nm, resulting in the surrounding refractive index sensing accuracy of 2.8 × 10^−3^. Linear regression based on the cut-off wavelength *λ*_c_ of this TFBG in response to the surrounding refractive index *n*_SRI_ gives a response function *λ*_c_ = 513 *n*_SRI_ + 804.5. TFBGs with smaller tilt angles are expected to preserve this response function for spectral intervals centered at shorter wavelengths.

The classical approaches of TFBG inscription based on tilting the fiber with respect to the interference fringes cannot be used when it comes to tight focusing geometries that are required for through-the-coating inscription. Indeed, it was analytically shown that rotating either the phase mask or cylindrical lens in the plane of diffraction results in the split of the focal lines [31]. Even though the focal lines can be recombined by counter-rotating the phase mask and cylindrical lens, no tilted grating planes could be achieved inside the fiber core due to the intrinsic orthogonality of the recombined focal line and the interference fringes. In this respect, only ‘dynamic’ laser writing, i.e., writing that requires relative translation of the laser focus and fiber, should be employed for through-the-coating inscription of TFBGs. This is in contrast to the classical approaches that represent ‘static’ laser writing as they do not require any relative translation of the laser focus and fiber.

#### 2.3.4. FBGs with Ultra-Strong Cladding Modes

**Figure 10:**
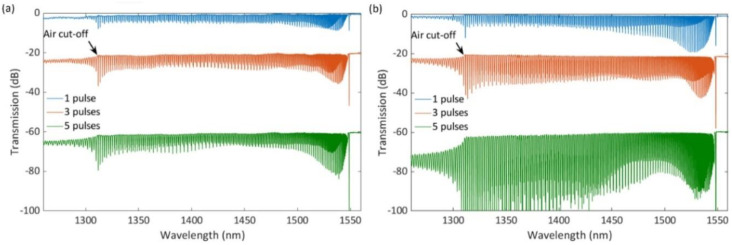
Transmission spectra of FBGs written using a different number of fs laser pulses. (**a**) laser beam polarization is perpendicular to the fiber axis. (**b**) laser beam polarization is parallel to the fiber axis. The spectra are plotted with an offset in the vertical axis for clarity.

It is not essential that tilted grating planes be inscribed in the fiber in order to create strong cladding modes. PbP-written FBGs whose grating points are offset with respect to the fiber axis, or core-offset gratings also exhibit strong coupling of the guided core mode into cladding modes [35,36]. It was demonstrated recently that FBGs with cladding modes that have a spectral span greater than 250 nm and are 30 dB-strong in transmission could be written with only a few high intensity fs laser pulses using the phase mask technique [37]. Using the same optical setup and optimal fiber positioning conditions, as discussed previously in this paper, single 800 nm 80 fs laser pulses were used to irradiate the optical fiber using the *Ibsen* phase mask. The resultant peak intensities within the fiber were kept at roughly two times the intensity threshold for optical breakdown in bulk fused silica [3]. Under these conditions, Abdukerim et al. fabricated FBGs with very strong cladding mode coupling (see Figure 10). Such FBGs can also be easily inscribed through the polyimide coating of the fiber. Because the coating suppresses higher order cladding modes, the FBG spectra presented in Figure 10 were obtained on bare fibers.

As it can be seen from Figure 10, laser beam polarization parallel to the fiber axis was more favorable for producing strong cladding modes than the beam polarization perpendicular to the fiber axis. For laser beam polarization parallel to the fiber axis, the cladding modes were at the level of 7–15 dB in transmission (depending on the wavelength) even in the case of single pulse irradiation, while five pulses with the same light intensity produced cladding modes in excess of 30 dB.

**Figure 11:**
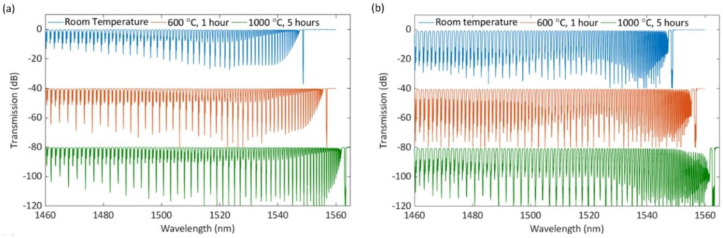
(**a**) High temperature performance of FBGs written with (**a**) 1 and (**b**) 5 pulses (laser beam polarization is parallel to the fiber axis).

As shown in Figure 11, the resulting devices are thermally stable up to 1000 °C and, in principle, can be used as a combined temperature/strain sensor at high temperatures [38].

#### 2.3.5. TTC-Written FBGs for Environmental Sensing Applications (Optical Trigger)

If a transductive layer or package is applied to an FBG, one that could convert a desirable measurand into a strain, the FBG would then act as a sensor for that measurand. Recently, for example, an FBG was embedded within a magnetostrictive polymer composite comprising epoxy resins and Terfenol D in order to create an FBG sensor sensitive to magnetic fields [39]. The package, upon exposure to a magnetic field, compresses the FBG resulting in a detectable strain-induced Bragg wavelength shift. For environmental applications, FBGs have also been demonstrated for detection of hydrocarbons [40]. FBGs photo-inscribed using a UV laser were packaged in a polymeric material that would act as a transduction element by swelling upon exposure to hydrocarbons. The swelling caused a tensile strain in the fiber which was detected by the FBG. However, the traditional UV photo-inscription procedure requires stripping and recoating of the fiber along with the complex packaging geometry, which significantly reduces the reliability of such a sensor especially when subjected to a tensile strain. On the other hand, TTC-written Type I FBGs can withstand strain levels similar to that of the pristine fiber [25,26].

Here, a FBG array for environmental sensing applications is manufactured in single mode telecommunications optical fiber through the protective polyimide coating using tightly focused fs IR laser pulses. By exploiting the high mechanical reliability of FBGs fabricated using TTC inscription with fs IR lasers, a robust FBG sensor that produces a trigger signal when exposed to hydrocarbons (e.g., crude oil) can be fabricated. The response is much stronger than that produced by thermal or acoustic effects which are normally used for fiber optic sensors applied to oil pipeline leak detection.

The method used to create the TTC-FBGs has been described previously [23]. Using the experimental conditions given in Section 2.3, TTC-FBGs were written into polyimide-coated SMF-28-type optical fiber using the *Ibsen* phase mask and an acylindrical lens with a focal distance *f* = 15 mm. The interplay of chromatic, aberration and conical diffraction effects caused by the phase mask and the focusing optics was accounted for by placing the fiber at *L*~300 μm away from the phase mask where the confocal parameter of the laser focus was smallest and, thus, the peak intensity in the focus highest [20]. This also ensured large intensity differentials between the fiber core and the protective coating which are required for TTC inscription of FBGs. Using these exposure conditions, TTC-FBGs were written in the Type I regime [34] where higher mechanical reliability was observed [25,26].

**Figure 12:**
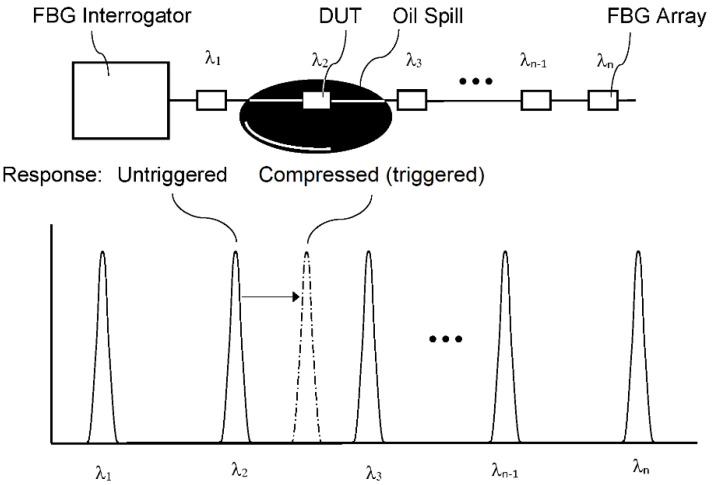
Schematic of a WDM-based interrogation system used to detect liquid hydrocarbons such as crude oil. *λ*_1_…*λ_n_* denote the respective Bragg wavelengths of the DUTs.

The concept of a leak detection system based on a wavelength demultiplexing approach (WDM) is presented in Figure 12. A commercially available FBG interrogator is used to probe a quasi-distributed FBG array of specially packaged sensor elements. The device under test (DUT) exposed to a liquid hydrocarbon, such as crude oil, undergoes a trigger response leading to a shift of the Bragg wavelength.

**Figure 13:**
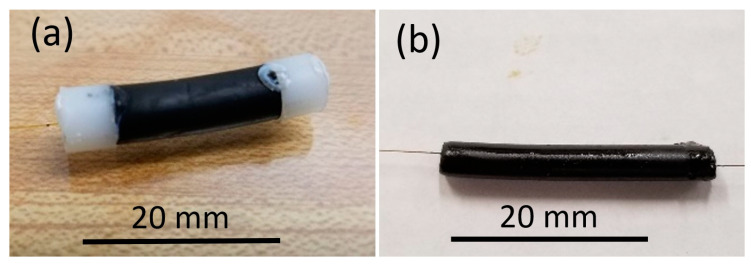
EPDM TTC-FBG packages (**a**) with polystyrene anchors (A) and (**b**) without anchors (B).

The packaging geometry considered here is similar to that of Spirin et al. [40]. In our case, a polyimide-coated TTC-FBG with 10% reflectivity is packaged with an elastomeric cord (e.g., ethylene propylene diene monomer rubber (EPDM)) that is end-capped with thermally molded polystyrene (PS) ferrule anchors to transfer stress caused by swelling in the cord to the fiber. EPDM will expand when exposed to crude oil but neither EPDM nor PS will react upon exposure to water. A more compact configuration of this packaging is also demonstrated where no PS ferrule anchor is required and instead the EPDM cord is simply glued to the polyimide fiber coating using Loctite 495 cyanoacrylate glue. When either package type is exposed to oil, a positive shift in the Bragg wavelength is observed. Images of both packaging styles are presented in Figure 13.

**Figure 14:**
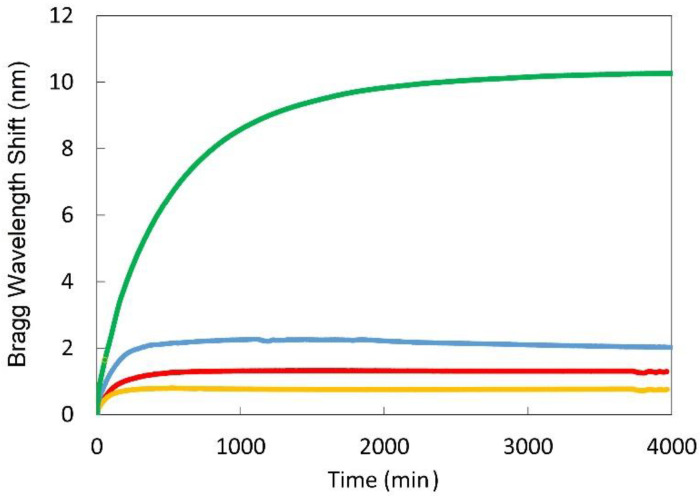
Bragg wavelength shift of EPDM packaged TTC-FBGs vs. time in crude oil. The green curve corresponds to the Bragg wavelength shift of package A with 6.4 mm EPDM cord and PS anchors; package B sensors with 1.6 mm, 2.4 mm, and 3.2 mm EPDM cord diameters and Loctite adhesive only are denoted by the yellow, red and blue traces, respectively.

Both A and B packaging geometries were tested in crude oil (Access Western Blend) at room temperature. The strain-induced shifts of the Bragg wavelength reflection peaks were measured every 60 s using a *Micron Optics* SM125 fiber Bragg grating interrogator with a 1 pm wavelength resolution (see Figure 14). The magnitude of the positive Bragg wavelength shift, indicative of a tensile strain applied to the fiber when immersed in oil, was dependent upon the EPDM package diameter and whether or not the PS anchors were present to amplify the tensile strain. As PS is resistant to degradation when exposed to crude oil, it made a suitable anchor material for these tests. For package A with a ~6.4 mm outer diameter of the EPDM package, very large amounts of strain were observed. Maximum wavelength shifts occurred after 30 h of immersion in oil. For the unanchored package B, the time for maximal Bragg wavelength shift was also dependent on the package diameter and occurred after 100 or 300 min for ~1.6 mm and ~3.2 mm cord diameters, respectively.

Temperature sensitivity of the package B type sensors were measured from 24 °C to 80 °C. The Bragg wavelength shifts of the sensors were found to be 0.04 nm/°C and 0.06 nm/°C for the 1.6 mm- and 3.2 mm-package devices, respectively. As the Bragg wavelength shifts due to temperature or immersion in oil are of the same order of magnitude, an unpackaged grating could be placed adjacent to the EPDM sensor as a temperature reference. No significant variation in the Bragg wavelength was observed in both types of packages when the devices were immersed in water at room temperature for 24 h.

## 3. Femtosecond Laser-Enhanced Fiber Scattering: Random Fiber Gratings

In addition to fabrication of periodic FBG structures, fs lasers can also be used to create quasi-randomly spaced grating planes using a variant of the PbP method known as the plane-by-plane technique [41]. With this approach, random fiber grating structures (RFG) can be created. The concept of the RFG was first theoretically studied by Derevyanko [42] where segmented pseudo-random modulation of the refractive index profile of a fiber Bragg grating resulted in a disordered structure. It was found that if the segments were larger than the correlation length, it would be possible to achieve a flat-top refection spectrum. This concept was experimentally demonstrated by employing phase noise and pseudorandom variations of the refractive index to successfully fabricate a flat-top FBG response [43] Such random fiber gratings (RFG) have been used in distributed temperature measurements [9,44,45], fiber lasers [46,47,48] and fiber laser sensors [49,50,51] over the past 5 years. The pertinent applications also include structural health monitoring, harsh environment sensing [45], perimeter intrusion detection and encrypted communication [52].

Rayleigh backscattering in optical fibers has been used for fiber sensing and fiber laser applications for decades [46,53,54,55,56]. In telecommunications single mode fibers (e.g., SMF-28 from Corning), the Rayleigh backscattering signal is usually very low (−100 dB/mm) as the fiber is designed for long-haul applications with minimized scattering loss. When such fibers are used for distributed sensing, the low backscattering signal level limits the sensitivity and spatial resolution of distributed temperature or stain measurements. When fiber with low Rayleigh backscattering is used as the distributed feedback for a random fiber laser application, a long piece of fiber is required resulting in a high lasing threshold and poor stability. In recent years, UV lasers have been used to increase light backscattering in optical fibers for distributed temperature and strain measurements with higher spatial resolution and higher signal-to-noise ratio (SNR) [44,57]. However, H_2_-loading of SMF-28 and specialty fibers with high Ge-concentrations are usually required to increase the fiber’s UV photosensitivity. Considering IR lasers, a CO_2_ laser was used to produce randomly spaced index changes in optical fiber for distributed feedback of fiber lasers with narrower linewidth and lower noise [47,48]. Fs IR lasers were also used to enhance backscattering in optical fiber for high temperature measurement [45]. In these applications, the IR laser-induced very high broadband losses.

### 3.1. Femtosecond Laser Fabrication of RFGs

Recently, we significantly expanded the use of fs IR lasers to fabricate RFGs in standard single mode fiber (SMF-28, Corning) for distributed sensing. The varieties of RFGs include ultra-low insertion loss RFGs for long-haul distributed sensors based on fiber lasers, broadband RFGs for multi-parameter sensing, and strong RFGs for the use in fiber lasers and fiber laser sensor applications.

**Figure 15:**
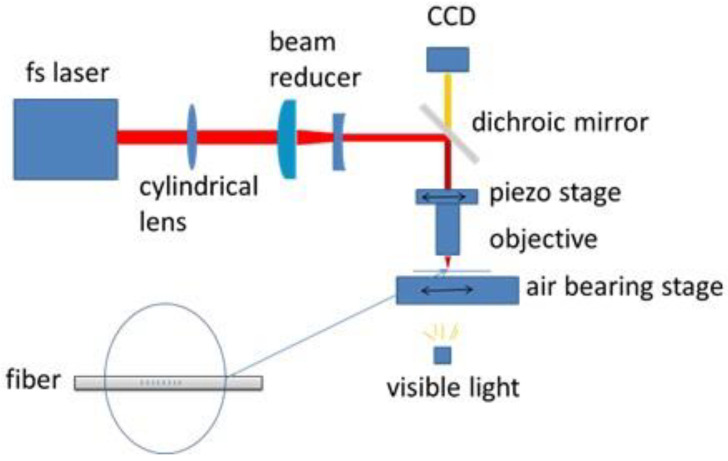
A schematic of the setup used to fabricate RFGs.

The setup of the plane-by-plane inscription of RFGs is similar to what was shown previously to write standard FBGs [41] (see Figure 15). A fs IR beam from a Ti:sapphire regenerative amplifier (Spitfire, Spectra-Physics) was focused onto the fiber core with a microscope objective (50×, NA = 0.6). To increase the overlap of the laser-induced index change and the modal field in the fiber core, a cylindrical lens with a focal length of either 0.5 m or 1 m was introduced into the laser writing setup before the microscope objective. The cylindrical shape of the focal volume normally produced by the microscope objective alone is instead transformed into a planar strip. The fiber was clamped to a precision air-bearing stage (Aerotech) with a ±10 nm position accuracy. The fs IR laser operated at a central wavelength of *λ*_0_ = 800 nm with a repetition rate up to 1 kHz and with a pulse duration of ~120 fs. The microscope objective was mounted on a translation stage (driven by a piezo actuator) that could move the microscope objective back and forth along the fiber by up to 20 μm. The fiber coating was removed so that the laser beam could be easily focused onto the fiber core. The fiber was free-standing, with no index matching fluid used between the fiber and the objective.

The plane-by-plane approach has certain advantages over the point-by-point approach for the fabrication of RFGs and FBGs more generally. For the PbP approach, the dimensions of the index change of a grating ‘plane’ are typically much smaller than the core (<1 μm) resulting in poor overlap between the guided mode field propagating along the fiber core and the grating structure. To increase the coupling between the guided mode field and the grating, either the magnitude of the index change or its physical dimension needs to be increased. Although it is straight forward to increase the index change with the PbP approach, very high scattering losses are introduced as well. Several approaches have been reported to increase the overlap between the guided mode and the grating while producing lower scattering losses. Zhou et al. [58] demonstrated a scanning method to increase the index change dimension by inscribing a line rather than a point across the fiber core. After one line is made, the fiber is moved along the fiber axis and then another line of index change is made. Strong higher order FBGs were made with this technique but with very high scattering losses (~1.2 dB/cm). Williams et al. [59] presented a continuous core-scanning technique where a piezo stage was driven by a sine wave, resulting in a sinusoidal index change across the core. Strong first order FBGs with low induced scattering loss were fabricated with the approach. The fabrication process however is complicated, time consuming when long grating lengths are considered and requires the use of oil-immersion objectives.

The use of the long focal length cylindrical lens before the microscope objective, as shown in Figure 15, transforms the cylindrical shape of the focal volume normally produced by the microscope objective into a circular plane. Each plane can be easily inscribed with a single pulse in free-standing fiber without oil-immersion objectives, simplifying the grating fabrication. When intensities are used that result in Type I modification, very long (>250 mm) grating structures can be produced with very low losses (~0.02 dB/cm) [41].

**Figure 16:**
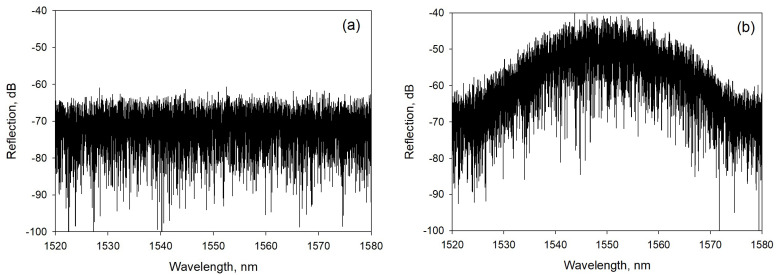
Simulated back reflection signals of two 100 mm long RFGs. (**a**) the grating period is randomly distributed from 0 to 2.5 μm and (**b**) the repetition rate of the fs IR laser is tuned randomly within a 2% range.

There are two approaches to fabricate RFGs using the setup shown in Figure 15. The selection of the particular approach depends on different application requirements. The first approach requires dithering of the piezo-driven stage along the fiber axis with random displacements while the air-bearing stage moves at a constant velocity and the laser operates at a constant repetition rate. Depending on the piezo stage used, this method can produce RFGs with random grating periods ranging from zero to a few micrometers. The second approach to fabricate RFGs requires the introduction of random changes to the laser repetition rate while the air-bearing stage moves at a constant speed and the piezo actuator is stationary. Details of these two methods are discussed elsewhere [9]. The first method involving the piezo stage is used for fabrication of RFGs with broadband spectral responses of up to a few micrometers which are useful for (i) multi-parameter sensors, (ii) fiber lasers and (iii) fiber laser sensors [49,50,60,61]. The second method, in which the piezo stage is not involved, is used to make (i) RFGs with narrow bandwidths, (ii) RFGs with low insertion losses for long-haul fiber sensor systems [9], and (iii) very strong RFGs for low-threshold fiber lasers to be used in ultrasound sensing with high sensitivity at frequencies in the MHz range [51]. Simulations presented in Figure 16 were performed using coupled mode theory and show the difference in spectral responses of RFGs fabricated using the two above methods. In both simulations, the laser induced index modulation was 1 × 10^−6^. The total grating length was 100 mm and comprised 4000 sub-gratings, each sub-grating with a uniform pitch that is 25 μm in length but with grating periods that varied randomly. In Figure 16a, the period of each sub-grating was distributed randomly between 0 and 2.5 μm. These conditions corresponded to the case where the air-bearing stage traveled at a constant velocity of 0.5 mm/s, the laser operated at 1 kHz repetition rate, and the piezo stage dithered with random displacements from 0 to 2 μm every 50 ms. In Figure 16b, the grating period of each sub-grating was randomly distributed from 0.5328 to 0.5436 μm, corresponding to the instance where the air-bearing moved at a constant velocity of 0.5382 mm/s and the repetition rate of the laser is randomly tuned every 50 ms from 990 to 1100 Hz (2%).

### 3.2. Applications of RFGs

Similar to FBGs, RFGs have been used widely in optical fiber communication and sensing applications. Here only the sensing applications of RFGs will be discussed.

#### 3.2.1. Distributed Sensing

**Figure 17:**
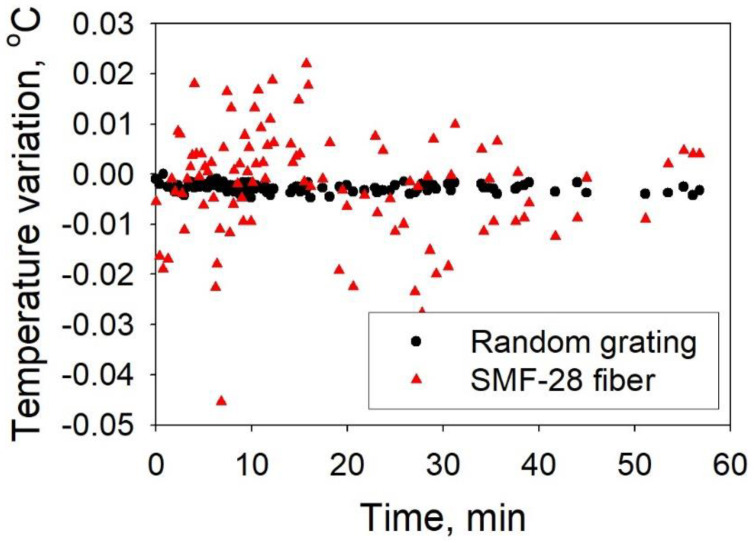
Variations of temperature measurement (error) when an RFG and regular SMF-28 were used to measure the water temperature in a re-circulating chiller/water bath where the water temperature was precisely controlled with temperature errors < 0.01 °C.

By using the fs IR laser frequency tuning method, RFGs with low laser-induced losses were fabricated in SMF-28 and used for distributed temperature measurements using the technique of optical frequency domain reflectometry (OFDR) [9]. The backscattering spectrum of the RFG was first measured as a reference, after which temperature-induced variations of the backscattering spectrum were measured and compared to the reference. The distributed spectral shift could then be obtained using a cross-correlation algorithm. To evaluate the sensor performance experimentally, a piece of 100 mm long RFG was submerged into a re-circulating chiller/water bath where the water temperature was precisely controlled and maintained at a constant temperature of 20 °C ± 0.01 °C. The backscattering spectrum of the RFG was repeatedly recorded every minute by an OFDR device (OBR4600, Luna) to monitor the temperature variation of the sensing system (measurement error). For comparison, the temperature measurement error was also simultaneously measured using a length of SMF-28 (see Figure 17). The advantage of using an RFG is obvious. With a gauge length of 10 mm, the standard deviation of the temperature measurement error was as low as 0.00085 °C in the case of RFG, while it was more than 0.01 °C in the case where SMF-28 was used. The laser-induced loss in this RFG is very low (0.08 dB/m), which is desired for long-haul fiber sensing networks.

#### 3.2.2. Multi-Parameter Sensing Based on RFGs

**Figure 18:**
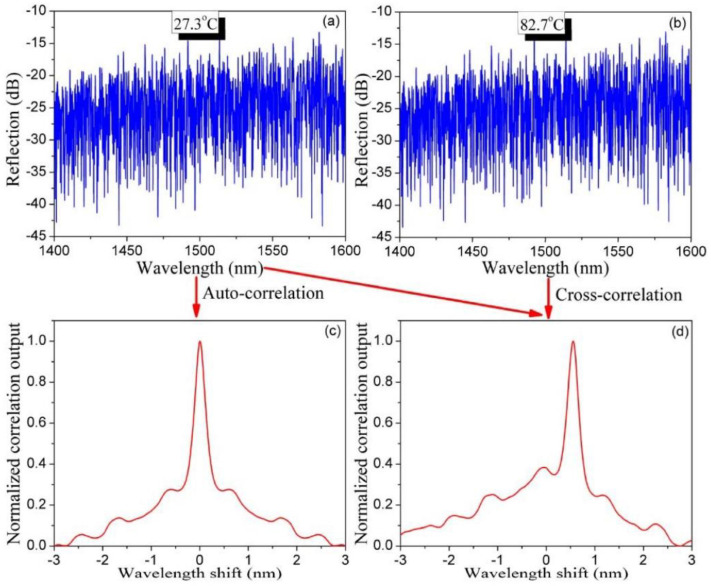
Measured reflection spectra of the RFG at (**a**) a reference temperature and (**b**) at an increased temperature; (**c**) the auto-correlation spectrum of (**a**,**d**) the cross-correlation spectrum of (**a**,**b**) (reproduced from [60]).

RFGs can be directly used for multi-parameter sensing [60]. For this application, an RFG was fabricated in SMF-28 with the piezo stage dithering method and with the fs IR laser pulse energy above the threshold of making Type II FBGs [34]. Due to the Type II nature of index change in the fiber, there is strong coupling between the fundamental propagation core mode and high-order cladding modes resulting in both a Mach–Zehnder interference (MZI) scheme via core-cladding mode coupling and a Fabry–Pérot interference (FPI) scheme via multi-reflected core-to-core mode coupling. The core-cladding mode coupling makes the grating spectral signature sensitive to surrounding refractive index change. Due to the waveguide and material dispersion effects in optical fiber, the grating spectral shifts caused by the changes in temperature, strain and surrounding refractive index have different dependencies on wavelength, which can be applied for multi-parameter sensing. It is not possible to observe spectral changes from the entire RFG by conventional peak detection methods typically used for FBG interrogation. Instead, a wavelength-division spectral cross-correlation algorithm is adopted to extract the spectral shift associated with different external disturbances. An autocorrelation of the reflection spectrum is performed which creates a correlation peak that is located at the center of the spectral range. When subjected to an external parameter such as a change in temperature, a shift in the correlation peak is observed. In Figure 18, two reflection spectra from the RFG are obtained, one at 27 °C (Figure 18a) and the other at 83 °C (Figure 18b). Reflection spectra were obtained using an optical spectrum analyzer with a 20 pm spectral resolution. Autocorrelation and cross-correlations of the two reflection spectra are presented in Figure 18c,d respectively.

**Figure 19:**
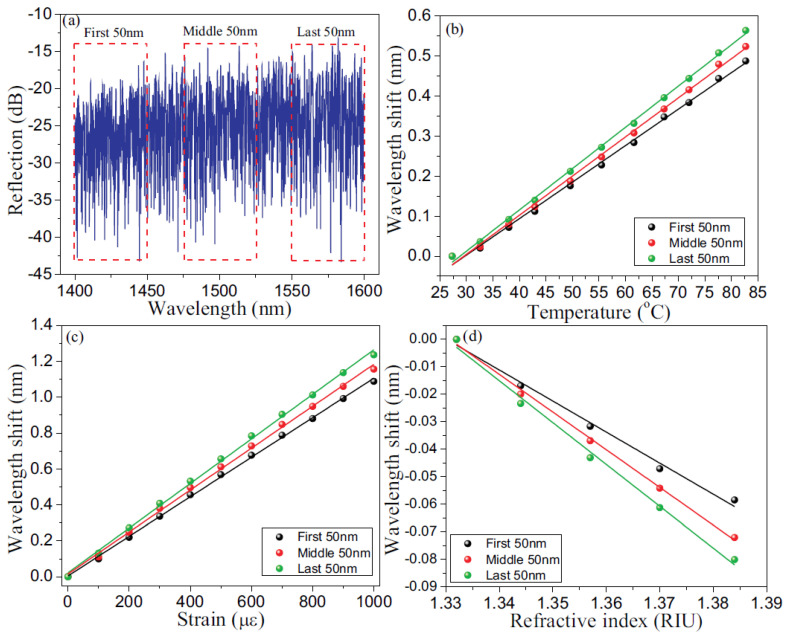
(**a**) Three spectral regions selected in the reflection spectrum of an RFG. Calibration results based on cross-correlation peak wavelength shift for (**b**) temperature, (**c**) strain, and (**d**) refractive index.

The cross-correlation peak wavelength is seen to shift with temperature. To simultaneously measure multiple parameters different wavelength regions of the broad bandwidth of the RFG are considered (see Figure 19a). The resulting cross-correlation wavelength shifts have different dependencies to external disturbances due to material and waveguide dispersion of the fiber allowing for the discrimination of changes to temperature, strain and surrounding refractive index (see Figure 19b–d).

According to the error analysis for simultaneous multi-parameter measurements [61], the minimum errors of temperature measurements for the three subsections respectively were 0.16 pm/°C, 0.16 pm/°C and 0.12 pm/°C. Similarly the axial strain errors are 0.01, 0.02 and 0.02 pm/με. The minimum errors of the RI measurement were 59.0, 37.7 and 65.8 pm/RIU.

**Figure 20:**
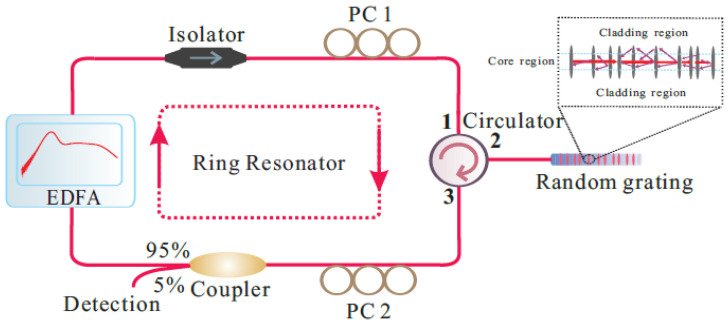
A schematic diagram of the experimental setup of the RFG-based Erbium-doped fiber ring laser; the magnified call-out shows a schematic of light backscattering within an RFG. EDFA and PC stand for Erbium-doped fiber amplifier and polarization controller.

**Figure 21:**
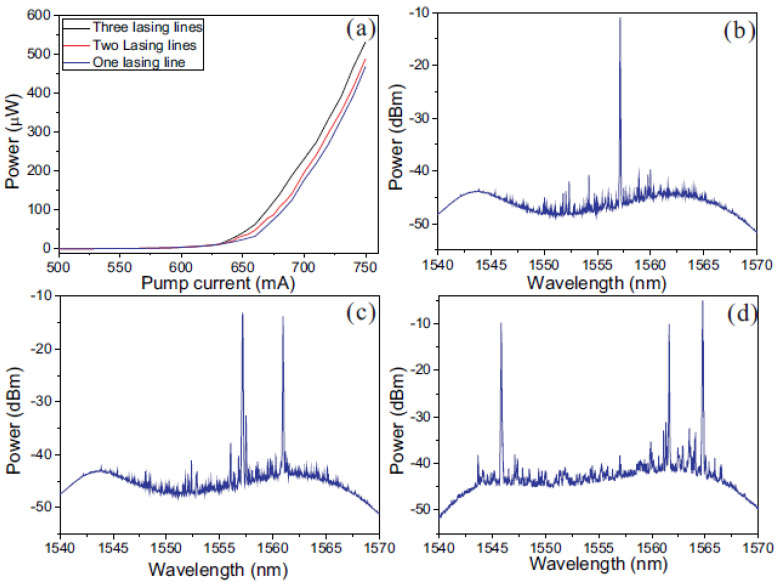
(**a**) Lasing thresholds for different numbers of emitted lasing lines as a function of pump current. Lasing spectra with different input polarizations are shown for (**b**) one lasing line, (**c**) two lasing lines, and (**d**) three lasing lines.

Multi-parameter sensing can also be realized by measuring the wavelength shifts of a random fiber laser where an RFG is used for distributed feedback [49]. Depending on the polarization of the light backscattered from the RFG and the pump current, an RFG-based random fiber laser can lase at multiple wavelengths. The lasing lines have different strain and temperature dependencies due to the different index modifications as well as dispersion effects. By monitoring the shifts of different lasing lines, simultaneous multi-parameter measurements could be achieved with better resolution compared with the single-pass random grating sensor. Figure 20 shows an experimental setup of a random fiber laser with an RFG incorporated for distributed feedback. Figure 21 shows the corresponding laser output with a different number of lasing lines that can be produced by adjusting the two polarization controllers in the setup along with the laser pump current. The number of sensing parameters can be controlled by the number of lasing lines.

#### 3.2.3. High Frequncy Ultrasound Sensing Based on RFGs

**Figure 22:**
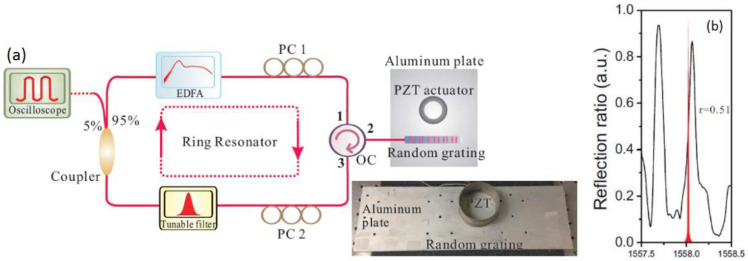
(**a**) A schematic of the RFG-based random laser sensor for ultrasound detection. (**b**) laser wavelength shift due to ultrasonic waves. PC1 and PC2 denote polarization controllers.

In this application, an RFG was not only used as a distributed feedback element for the random fiber laser, but also as a sensing element for ultrasound detection [50]. The RFG as well as a piezoelectric transducer (PZT) actuator were mounted onto an aluminum plate. To generate ultrasonic waves, the PZT actuator was driven by a function generator to produce Rayleigh waves that propagated as acoustic surface waves along the aluminum plate (see Figure 22a). The detection of the ultrasound was performed by monitoring the laser wavelength shift due to the ultrasonic wave, as shown in Figure 22b. The tunable filter shown in Figure 22a was set to select a specific laser line. Lasing of the erbium fiber ring laser shown in Figure 22a is possible at several wavelengths depending on the state of polarization of the intra-cavity light and the birefringent properties of the RFG [48]. A tunable filter is introduced to ensure single-wavelength lasing and the lasing line is selected such that the laser emission (red line in Figure 22b) rests on the steep slope of one of many reflectivity peaks generated by the RFG (black line in Figure 22b). The dynamic strain generated by the PZT actuator results in a shifting of the RFG reflection spectrum which causes a modulation of the lasing output power. The proposed laser sensor exhibited a broadband ultrasound response within a 0.8 MHz frequency range with a high SNR.

#### 3.2.4. Random Fiber Lasers

Besides the numerous applications in sensing, RFGs have also been used to significantly improve the performance of random fiber lasers resulting in fiber lasers with new features. We will not discuss all the details here but only provide a brief summary of the pertinent results: (i) low frequency noise Brillouin random fiber laser based on an RFG feedback was demonstrated with a very narrow laser line width (<50 Hz) [62], (ii) the time-delay signature of an RFG-based fiber laser was suppressed to be as low as 0.0088 ± 0.0015 [63], which is of extreme importance for real-time physical random bit generation [52], (iii) multi-wavelength Brillouin random fiber lasers [64] and thermal/acoustic noise insensitive Brillouin random fiber lasers [65] were realized due to the incorporation of RFGs into their design.

## 4. Conclusions

In this review, we presented recent advances in TTC femtosecond laser inscription of FBGs and how it relates to sensing applications. Namely we showed that TTC fabrication of distributed FBG arrays in 50 m diameter fiber filaments becomes possible by using a femtosecond laser and the phase mask technique if the complex diffraction and dispersion effects highlighted in this article are properly taken into account. Such FBG sensor arrays can be easily integrated into composite materials for structural health monitoring or 3D printed components for smart structures. We also showed that femtosecond lasers and the phase mask technique can be used for TTC fabrication of tilted or core-offset gratings for multi-parameter or refractive index sensing. The specific application of a specially packaged TTC-written FBG for distributed oil pipeline leak detection is also presented.

In addition, this article reviews recent developments in fs laser fabrication of RFGs and applications of the resultant RFGs. More specifically, we demonstrated that the spectrum of an RFG inscribed using a femtosecond laser and the plane-by-plane technique can be easily tailored for different applications, such as highly accurate distributed temperature/strain measurements, high sensitivity multi-parameter sensing, and high-frequency ultrasound detection.
